# PIANO: A Web Server for Pseudouridine-Site (Ψ) Identification and Functional Annotation

**DOI:** 10.3389/fgene.2020.00088

**Published:** 2020-03-12

**Authors:** Bowen Song, Yujiao Tang, Zhen Wei, Gang Liu, Jionglong Su, Jia Meng, Kunqi Chen

**Affiliations:** ^1^ Department of Biological Sciences, Xi'an Jiaotong-Liverpool University, Suzhou, China; ^2^ Institute of Integrative Biology, University of Liverpool, Liverpool, United Kingdom; ^3^ Institute of Ageing & Chronic Disease, University of Liverpool, Liverpool, United Kingdom; ^4^ Department of Mathematical Sciences, Xi'an Jiaotong-Liverpool University, Suzhou, China

**Keywords:** pseudouridine sites, genome-derived feature, RNA modification, Web-server, functional annotation

## Abstract

Known as the “fifth RNA nucleotide”, pseudouridine (Ψ or psi) is the first-discovered and most abundant RNA modification occurring at the Uridine site, and it plays a prominent role in a number of biological processes. Thousands of Ψ sites have been identified within different biological contexts thanks to the advancement in high-throughput sequencing technology; nevertheless, the transcriptome-wide distribution, biomolecular functions, regulatory mechanisms, and disease relevance of pseudouridylation are largely elusive. We report here a web server—PIANO—for **p**seudouridine site (Ψ) **i**dentification **a**nd fu**n**ctional ann**o**tation. PIANO was built upon a high-accuracy predictor that takes advantage of both conventional sequence features and 42 additional genomic features. When tested on six independent datasets generated from four independent Ψ-profiling technologies (Ψ-seq, RBS-seq, Pseudo-seq, and CeU-seq) as benchmarks, PIANO achieved an average AUC of 0.955 and 0.838 under the full transcript and mature mRNA models, respectively, marking a substantial improvement in accuracy compared to the existing *in silico* Ψ-site prediction methods, i.e., PPUS (0.713 and 0.707), iRNA-PseU (0.713 and 0.712), and PseUI (0.634 and 0.652). Besides, PIANO web server systematically annotates the predicted Ψ sites with post-transcriptional regulatory mechanisms (miRNA-targets, RBP-binding regions, and splicing sites) in its prediction report to help the users explore potential machinery of Ψ. Moreover, a concise query interface was also built for 4,303 known Ψ sites, which is currently the largest collection of experimentally validated human Ψ sites. The PIANO website is freely accessible at: http://piano.rnamd.com.

## Introduction

Pseudouridine (5-ribosyluracil, Ψ, and psi) is the first-discovered (Cohn and Volkin, 1951) and most abundant RNA modification occurring at the Uridine site catalyzed by 13 pseudouridine synthase (PUS) (Chen and Patton, 2000; Zhao et al., 2004; McCleverty et al., 2007; Shaheen et al., 2016; Jacob et al., 2017). Ψ is present in many classes of RNA within all organisms, such as messenger RNA (mRNA), transfer RNA (tRNA), small nucleolar RNA (snoRNA), small nuclear RNA (snRNA), and ribosomal RNA (rRNA) (Ge and Yu, 2013). Ψ was termed as “the fifth nucleotide” with an estimated Ψ/U ratio of 7–9% (Jacob et al., 2017), and it is considered to be the most prevalent of the mRNA modifications (Meyer and Jaffrey, 2017). Ψ plays a prominent role in many biological processes. The presence of Ψ in tRNA and rRNA regulates the entry site binding process in ribosomal RNA (Jack et al., 2011) and RNA structure stabilization (Kierzek et al., 2014). A recent study also demonstrated that Ψ is related to transcript stability (Schwartz et al., 2014), environmental signal response (Carlile et al., 2014), and genetic code switching in mRNA (Karijolich and Yu, 2011; Fernández et al., 2013). Ψ deficiency may be associated with various diseases. It has been found that the dysregulation of Ψ modification of mitochondrial tRNA acts as an etiology of mitochondrial myopathy and sideroblastic anemia (MLASA) (Bykhovskaya et al., 2004). Furthermore, mutations in pseudouridine are also involved in diseases like lung cancer and duykeratosis congenita (Mei et al., 2012).

Several high-throughput sequencing approaches have been developed for profiling the transcriptome-wide distribution of Ψ, including Pseudo-seq (Carlile et al., 2014), Ψ-seq (Schwartz et al., 2014), PSI-seq (Lovejoy et al., 2014), and CeU-seq (Li X,et al., 2015). These approaches all share the same principle, in which RNA is treated with the N-cyclohexyl-N’-(2-morpholinoethyl)-carbodiimide-metho-*p*-toluenesulfonate (CMC) to leave a bulky group on Ψ and stop reverse transcription. Since the bulky adduct on the Ψ may reduce the sensitivity in the detection of Ψ, Vahid et al. recently developed a new approach, RBS-seq, which is based on a modification of RNA bisulfite sequencing and claims better sensitivity (Khoddami et al., 2019). Currently, the experiment-validated Ψ sites in human, mouse, and a few other model organisms are available from RMBase database (Xuan et al., 2017), and the regulation pathways of Ψ were more explicitly explained in MODOMICS database (Boccaletto et al., 2017).

Wet-lab approaches are surely effective for the study of transcriptome pseudouridylation with respect to a specific biological context; however, they are also laborious and offer only limited coverage, i.e., the reported RNA Ψ sites by wet-lab experiments are still restricted to the transcripts more readily expressed under a specific cell/tissue condition. Alternatively, computational efforts may provide a more cost-effective avenue (Chen X, et al., 2017). To date, many computational efforts have been made to facilitate the study of RNA epigenetics (Boccaletto et al., 2017; Chen X, et al., 2017; Chen Z, et al., 2019; Xue et al., 2020; Liu et al., 2020) in terms of both experimental data collection and site prediction works. For predictors related to the identification of Ψ RNA modification, PseUI (He et al., 2018), XG-PseU (Liu et al., 2019), and iRNA-PseU (Chen et al., 2016) allow for prediction of putative Ψ sites from an RNA sequence, and PPUS (Li Y.H, et al., 2015) can predict the Ψ sites regulated by a specific pseudouridine synthase. However, these three predictors are all based on sequence-derived features only without considering other genomic features (such as conservation, gene annotation, and miRNA binding) that may contribute to the prediction, and thus their performance is limited (Chen K, et al., 2019). Moreover, their prediction results are not functionally annotated with potential post-transcriptional regulation machineries that may explain the functional consequences of the predicted Ψ sites.

We present here a web server—PIANO—for **p**seudouridine site **i**dentification **a**nd fu**n**ctional an**n**otation. Inspired by the WHISTLE framework (Chen K, et al., 2019), PIANO took advantage of both the conventional sequence features and 42 additional genomic features. Using six independent datasets generated from four different technologies, we showed that PIANO adds a marked improvement to the accuracy of existing Ψ-site prediction. Moreover, the PIANO web server accepts both genomic location and RNA sequence format as input file when making predictions, and the putative Ψ sites returned are also annotated with various post-transcriptional regulations, including miRNA-targets, RBP-binding regions, and splicing sites, to unveil potential functional mechanisms of Ψ. The PIANO website is freely accessible at: http://piano.rnamd.com.

## Materials and Methods

### Training and Testing Data for Ψ-Site Prediction

To construct the Ψ-site prediction model, we used the known human Ψ sites detected from four different base-resolution Ψ profiling techniques, including Ψ-Seq, RBS-Seq, CeU-Seq, and Pseudo-Seq (see **Table 1**). The Ψ sites at base-resolution were directly downloaded from Gene Expression Omnibus (GEO).

**Table 1:** Base-resolution dataset used for Ψ-site prediction.

Dataset	Cell line	Treatment	Technique	Site #	Source
H1	HEK293		Ψ-Seq	652	(Schwartz et al., 2014)
H2	Hela		RBS-Seq	322	(Khoddami et al., 2019)
H3	HEK293T		CeU-Seq	1555	(Li X, et al., 2015)
H4	HEK293T	H_2_O_2_		460	
H5	HEK293T	Heat Shock (HS)		421	
H6	Hela		Pseudo-Seq	156	(Carlile et al., 2014)

The experimentally validated human Ψ sites used in this project are also available from the PIANO website of this project (http://piano.rnamd.com), annotated with various post-transcriptional regulations.

**Figure 1:**
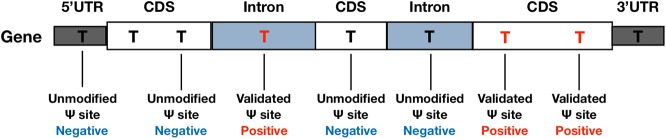
Negative and Positive Data. Negative sites were randomly selected from un-modified U sites located on the same transcripts of the positive sites.

In the beginning of the performance evaluation, dataset H1 (see **Table 1**) was used as the testing data, while dataset H2-H4 were used as for training. Specifically, the base-resolution Ψ sites in training datasets were used as the positive training data. The negative sites used in model training were randomly selected from unmodified U sites located on the same transcripts of positive sites (see **Figure 1**). To make the best use of the limited volume of positive data, we randomly selected 10 negative sites for each of the positive sites. To balance the positive-to-negative ratio, the negative sites were then randomly split into 10 subsets, and 10 separate predictors were generated with a 1:1 positive-to-negative ratio. The negative sites of testing data were generated following the same procedure. Consequently, 10 separate predictors were generated, and their prediction results were averaged.

Following the experimental design of WHISTLE framework (Chen K, et al., 2019), we performed dataset level leave-one-out validation over the H1-H5 base-resolution datasets; four samples from H1–H5 were used as training, while the other was used for testing. Subsequently, the sites from the datasets H1–H5 (generated from Ψ-Seq, RBS-Seq, and CeU-Seq) were used to establish a predictor, whose performance was evaluated on the dataset H6, which was generated from an independent technology (Pseudo-Seq).

### Features Used for Ψ-Site Prediction

#### Sequence-Derived Features

The length of 41bp was widely used to extracted sequence information in many previous studies, which was determined as a suitable flanking window by relevant tests, i.e., iRNA-m7G (Liu et al., 2019), iRNA-2OM (Yang et al., 2018), and MethyRNA (Chen W, et al., 2017). Consequently, the sequence-derived information of 41 bp flanking window of Ψ and non-Ψ (U) sites as central was generated using the chemical properties of nucleotides, position-specific nucleotide propensity (PSNP), and cluster information.

In the first encoding method, the nucleotides are classified into three categories based on three distinct structural chemical properties. Ring structures of nucleotides are the first to be considered; here, adenosine and guanosine have two rings, while cytidine and uridine only have one ring. In addition, the guanosine and cytidine have stronger hydrogen bonding than adenosine and uridine. Furthermore, adenosine and cytidine can be classified as the amino group, while guanosine and uridine contain the keto group. Based on these chemical properties defined above, the *i* -th nucleotide from sequence *S* may be encoded by a vector *S_i_* = (*x_i_*, *y_i_*, *z_i_*):

(1) $$x_{i}=\{{}_{{0 if s}_{i}\in \left\{\text{C},\text{U}\right\}}^{1{\text{ if s}}_{i}\in \left\{\text{A},\text{G}\right\}},\;y_{i}=\{{}_{{0 if s}_{i}\in \left\{\text{G},\text{U}\right\}}^{1{\text{ if s}}_{i}\in \left\{\text{A},\text{C}\right\}},\;z_{i}=\{{}_{{0 if s}_{i}\in \left\{\text{C},\text{ G}\right\}}^{1{\text{ if s}}_{i}\in \left\{\text{A},\text{U}\right\}}$$

Thus, the A, C, G, and U may be encoded as a vector (1,1,1), (0,1,0), (1,0,0), and (0,0,1), respectively.

The position-specific nucleotide propensity (PSNP) stands for the differences of the frequency of nucleotides calculated in specific locations between RNA sequences of positive and negative data. The frequency of occurrence of A, U, G, and C in the *i* -position were calculated for both positive and negative data, respectively, to obtain two matrices with 4×41 dimension as Z_plus_ and Z_minus_, where Z_plus_ was extracted from sequence of all positive data, and Z_minus_ was extracted from sequence of all negative data. The position-specific nucleotide propensity (PSNP) matrices was defined as Z_PSNP_:

(2) $$Z_{\text{PSNP}}=Z_{\text{plus}}=Z_{\text{minus}}$$

For the cluster information, the average relative position of the closest *k (k=1,2 and 3)* nucleotide to center Ψ/non-Ψ was calculated for each nucleic acid (A, G, C, and U). The *k* was considered as 1 to 3. Using sequence ‘AGCUAGCCAUCCUACGGUACAGCAU’ as an example, the center U is at the ninth positive. For encoding the cluster information of adenine, the average relative position of the closest 1 (k=1) adenine to center U is 1 (1/1); when k equals to 2, the relative position of the second closest adenine to center U is 4, and, therefore, the average relative position of the closest 2 (k=2) adenine to center U is 2.5 (5/2) and 3.7 (11/3) when k equals to 3. Similarly, the cluster information of guanosine in this example sequence is 3 (3/1), 3.5(7/2), and 4.7(14/3) when k equals to 1, 2, and 3, respectively.

The sequence-derived encoding methods employed by the three previously published predictors were used to reproduce the PPUS, iRNA-PseU, and PseUI with the same training data of PIANO, respectively, and their performances were compared with PIANO using independent datasets.

#### Genome-Derived Features

In the original WHISTE approach, 35 additional genomic features that might contribute to the prediction of m^6^A RNA methylation sites were considered (Chen K, et al., 2019). In PIANO, seven new genomic features were added to the prediction model, the details of the 42 genomic features considered in the prediction were summarized in **Supplementary Table S1**. Specifically, genomic Features 1–16 are dummy variable features indicating whether the uridine sites shall fall within the transcript regions that satisfy certain topological properties. All the features in this category are generated by the GenomicFeatures R/Bioconductor package using the transcript annotations hg19 TxDb package (Lawrence et al., 2013). To remove the ambiguity caused by transcript isoforms, only the primary (longest) transcripts of each gene were kept for the extraction of the transcript sub-regions. The longest transcript isoform was used to unambiguously assign m6A peak regions to mRNAs (Ke et al., 2017) and contributed to a better performance in accuracy compared with using the average value of multiple transcripts. Genomic Features 17–20 are real valued features defining the relative position of the transcript regions (3’UTR, 5’UTR, CDS, and whole transcript), i.e., the distance from the adenine to the 5’ end divided by the width of the region. The values are also set to zero for sites that do not belong to the region. Genomic features 21–25 represent the length of the transcript region containing the modification site. The values are also set to zero for sites that do not belong to the region. Features 26–27 captured the distance from the adenine sites to the 5’end or 3’end of the splicing junctions. Additionally, the distance to the nearest neighboring ψ sites in the training data is generated to measure the clustering effect of the ψ RNA modification sites. Evolutionary conservation score of the uridine sites and its flanking regions are measured by Phast-Cons (Siepel et al., 2005) score, and the fitness consequence (Gulko et al., 2015) scores were presented in features 28–31. To consider the RNA secondary structures around the uridine site, the RNA secondary structures are predicted using RNAfold from the Vienna RNA package (Lorenz et al., 2011) and presented in features 32–33. Genomic properties of transcripts containing the Ψ sites were presented in features 34–38. Finally, features 39–42 represent omics information, such as microRNA target sites (Chou et al., 2017) and HNRNPC binding sites (2012).

### Machine Learning Approach Used for Ψ-Site Prediction

As a high-efficiency machine learning algorithm in computational biology, the SVM (Support Vector Machine) has been widely applied in microRNA target prediction (Liu et al., 2010), protein phosphorylation prediction (Wong et al., 2007), and m^6^A RNA methylation site prediction (Chen W, et al., 2017). In this project, the R language interface of LIBSVM (Chang and Lin, 2011) was used to build our model with the radial basis function as kernel, and the other parameters were set at the default.

### Performance Evaluation of Ψ-Site Prediction

To evaluate the performance of PIANO, a 5-fold cross-validation was employed on training datasets using the SVM classifier, and the independent testing dataset was used to measure the final performance of PIANO. There is no overlap between the training sites and testing sites, as only the Ψ sites not previously used as training data were considered during performance evaluation; the performance evaluation result should thus directly reflect the capability of the algorithm to identify previously unknown Ψ sites. To evaluate the performance, the ROC (receiver operating characteristic) curve (sensitivity against 1-specificity) was used, and the area under ROC curve (AUROC) was calculated as the main performance evaluation metric.

### Estimate the Probability of Ψ

The likelihood ratio (LR) of a Ψ site is calculated to estimate the probability of Ψ RNA methylation:

(3) $$LR=\frac{P(observation|\text{Ψ})}{P(observation|\text{U})}$$

In the PIANO web server, a site was predicted to be a putative Ψ site if its predictive value was above 0.5 with a minimum LR value of 1. A site with a larger LR value suggests that it is more likely to be a Ψ site. The machine learning classifiers usually obtain the lowest empirical rate with the value of 0.5 as cutoff. The statistical significance of LR is assessed by an upper bound of the p-value, indicating how extreme the observed LR is among all the transcriptome U sites. It is calculated from the relative ranking of the putative Ψ sites among all the transcriptome U sites, i.e., if only 0.1% of U sites have a LR score larger than a specific U site, then the upper bound of the p-value of this site is 0.001. In the report of PIANO web server, a putative Ψ site is considered to be of high confidence if its LR within the top 0.5% of all transcriptome Us (corresponding to an upper bound of the p-value < 0.005) of all the transcriptome U sites, followed by medium confidence (0.005 < upper bound of the p-value ≤ 0.05) and low confidence (p-value > 0.05).

### Functional Annotation of Putative Ψ Site

The gene symbol, Ensembl gene ID, gene region, and gene type for each putative Ψ site were annotated using ANNOVAR package (Wang et al., 2010). Furthermore, we annotated the putative Ψ sites with three kinds of post-transcriptional regulation, including RNA-binding proteins (RBPs) regions, miRNA-RNA targets, and splicing sites. We first found the intersection between the computational predicted Ψ sites and POSTAR2-derived RBP binding regions (Zhu et al., 2018). For miRNA targets, we obtained the information from miRanda (Agarwal et al., 2015) and starBase2 (Li et al., 2013), and we found the Ψ sites within the miRNA targets regions to explore the potential influence of Ψ on miRNA-target interactions. Finally, we obtained the Canonical splice sites (GT-AG) from UCSC (Lawrence et al., 2013) annotations, 100 bp upstream region from 5’ splicing sites and 100 bp downstream region from 3’ splicing sites were extracted for the subsequent analysis of Ψ sites on splicing sites. The detailed information of the post-transcriptional regulation association analysis can be found in **Supplementary Table S2**.

## Results

Although the genome-derived features alone are already very effective for predicting Ψ sites, the best performance was achieved when the sequence features and genomic features were combined. Consequently, our PIANO predictor was established based on both the genome-derived features and sequence-derived features. When designing the encoding methods for sequence features used for the PIANO approach, the chemical properties of nucleotides, position-specific nucleotide propensity (PSNP), and cluster information were considered. We found that this combination (sequence and genomic features) achieved the best performance in accuracy compared with combining genome-derived features with other basic sequence encoding methods (i.e., one-hot encoding method).

The performance of the predictor was evaluated under two modes. For the full transcript mode, the positive and negative Ψ sites located in both exonic and intronic regions are all considered to construct the predictor. In the mature mRNA mode, only positive and negative Ψ sites located on mature mRNA transcripts are considered; this is because existing experimental datasets overwhelmingly relied on polyA selection in RNA-seq library preparation, and intronic Ψ sites are likely to be underrepresented in the data, which may lead to an over-estimation of accuracy under the full transcript mode.

To avoid potential over-fitting and to identify the most significant subset of genomic features, feature selection was implemented; the datasets H2–H5 were used as training data, while dataset H1 was used for the independent testing data. The relative importance of each genome-derived feature were measured by the Perturb method (Gevrey et al., 2003). According to the rank of importance, the top N most important features were reserved in the prediction and were evaluated with a 5-fold cross-validation. For the predictor under full transcript model, the top 17 genomic features led to the best predictor performance, with fitCons scores, exons containing stop codons, and number of exons as the top three most important genomic features for prediction. Similarly, the top 20 genome-derived features were selected under the mature mRNA model. The length of the mature transcript plays the most important role under this model, and the exons containing stop codons and an miRNA target won the second and third significance. Consequently, to obtain the most robust performance, only the top 17 and 20 genomic features were used under full transcript model and mature mRNA model for Ψ site prediction, respectively. Please see **Supplementary Figure S1** for more details.

We showed that the newly developed method PIANO substantially outperformed competing approaches on cross-validation (**Supplementary Table S3**) when tested on independent datasets (**Supplementary Table S3**) or benchmarked by an independent technique (**Supplementary Table S4**). To sum up, by testing independent datasets generated from four different Ψ profiling technologies (Ψ-seq, RBS-seq, Pseudo-seq, and CeU-seq), the newly developed method PIANO achieved an average AUC of 0.955 and 0.838 under full transcript and mature mRNA modes, respectively (see **Table 2**), representing a marked improvement compared to PPUS (0.713 and 0.707), iRNA-PseU (0.713 and 0.712), and PseUI (0.634 and 0.652).

**Table 2:** Performance evaluation of Ψ-site predictors.

Mode	Method	Benchmarking data (AUC)				Average AUC
		Ψ-Seq	RBS-Seq	CeU-Seq	Pseudo-Seq	
Full transcript	PIANO	0.957	0.978	0.914	0.972	0.955
	iRNA-PseU	0.679	0.727	0.721	0.708	0.713
	PPUS	0.700	0.721	0.724	0.705	0.713
	PseUI	0.631	0.710	0.610	0.585	0.634
Mature mRNA	PIANO	0.859	0.770	0.864	0.857	0.838
	iRNA-PseU	0.753	0.582	0.760	0.751	0.712
	PPUS	0.749	0.575	0.757	0.748	0.707
	PseUI	0.666	0.651	0.652	0.639	0.652

The table presents the performance of different Ψ site predictors achieved on independent human datasets with different technologies as a benchmark, and it is summarized from **Supplementary Table S3** and **S4**. Only the Ψ sites not previously used as training data were considered during performance evaluation, so the training sites and testing sites did not overlap. Because existing datasets overwhelmingly relied on polyA selection in RNA library preparation and intronic Ψ sites are likely to be underrepresented in the data, the performances were evaluated under two modes: full transcript and mature mRNA modes. In the mature mRNA mode, only positive and negative Ψ sites located on mature mRNA transcripts are considered, as previously described (Chen K,et al., 2019). Our new approach PIANO substantially outperformed competing approaches in accuracy.

The performance of the purposed predictor was further evaluated by separating the training and testing datasets between the cell type in which datasets H3–H5 generated from HEK293T were used for training, while datasets H2 and H6 from Hela were used for independent testing. Consistent with previous validation results, our method PIANO achieved a marked improvement in prediction accuracy compared with existing predictors, using the AUROC (area under ROC curve) and AUPRC (area under precision-recall curve) as an evaluation metric, when tested on independent dataset with a 1:1 positive to negative ratio (**Supplementary Table S5**) and 1:10 positive to negative ratio (**Supplementary Table S6**), respectively, suggesting the reliability of our newly proposed approach. Besides, the comparison between different algorithms indicated that SVM (Support Vector Machine) was a quite effective machine learning approach and achieved the best performance in our study (**Supplementary Table S5**). In addition, to further evaluate different approaches, we also considered the prediction of PUS-specific Ψ sites. In this experiment, TruB1, PSU7, and TruB2 were considered, and the goal was to predict their specific substrates (Safra et al., 2017). Consistent with previous results in Ψ-site prediction, the PIANO method again substantially outperformed competing approaches under both the full transcript and mature mRNA model (**Table 3**), suggesting the effectiveness of the approach.

**Table 3:** PUS-specific substrate prediction.

Method	Full transcript model			Mature mRNA model		
	TruB2	PSU7	TruB1	TruB2	PSU7	TruB1
PIANO	0.981	0.966	0.973	0.837	0.960	0.910
iRNA-PseU	0.812	0.829	0.838	0.719	0.812	0.731
PPUS	0.806	0.824	0.824	0.733	0.816	0.739
PseUI	0.853	0.870	0.840	0.805	0.861	0.786

### Construction of the PIANO Website

**Figure 2:**
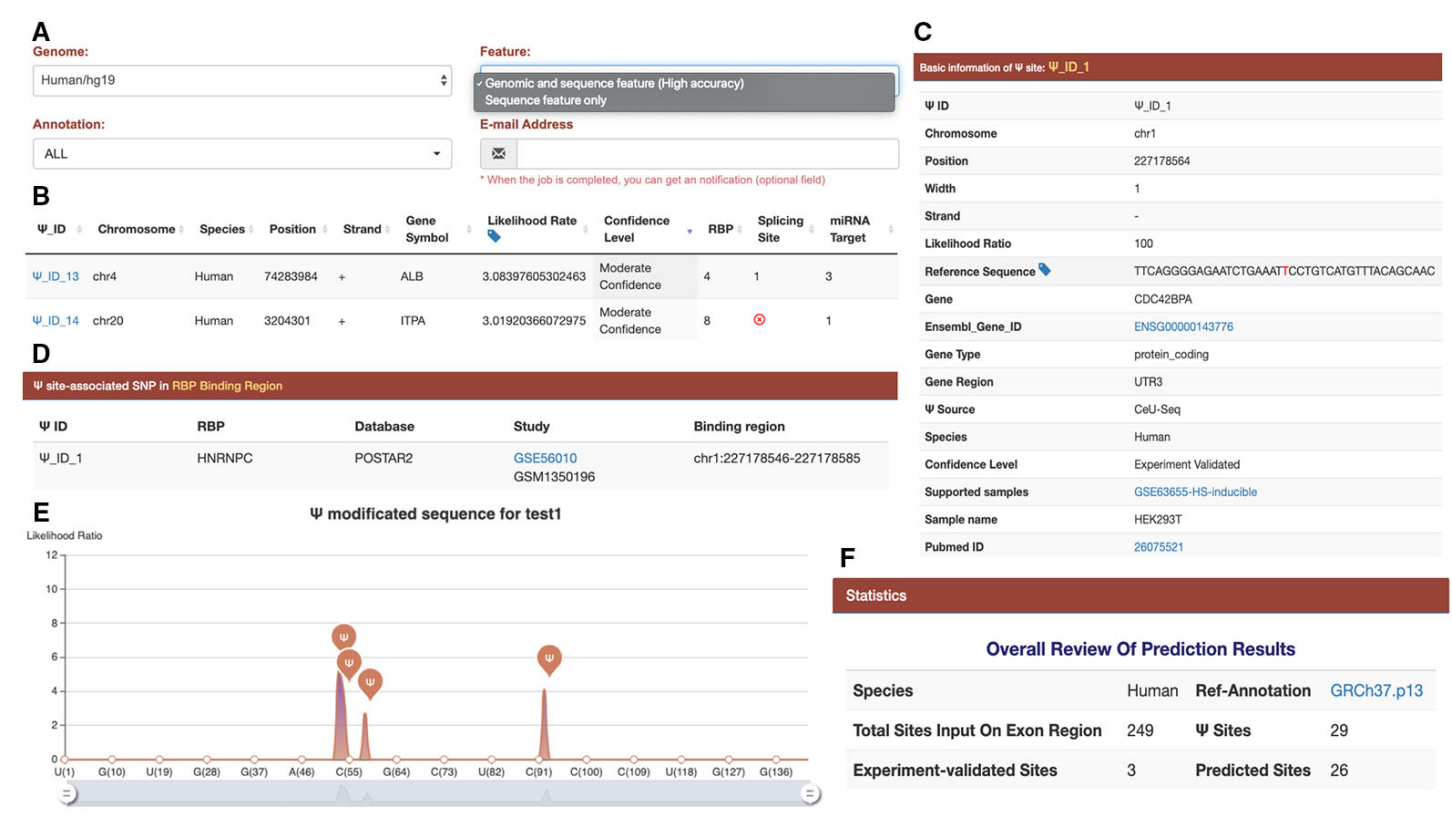
Interface and output of the PIANO web server for Ψ-site prediction and functional annotation. **(A)** When predicting human Ψ sites, the PIANO web server supports two types of input: the genomic ranges of human genome assembly and the FASTA sequences. As the prediction process may take quite some time, it is highly recommended that the user should provide an email address, where an email notification will be sent when the job is finished. **(B)** The basic information of each putative Ψ site, such as gene symbol, likelihood ratio, confidence level, and the number of related post-transcriptions associated with the putative site. **(C)** The source and detailed information of each putative Ψ site. If the input file contains any experimental validated Ψ sites collected in PIANO, the sites will be annotated with additional information. **(D)** The details of the site-relevant RBP information. **(E)** A graph to visualize the position of predicted Ψ sites on a user-provided FASTA sequence. **(F)** An overall review of the prediction result.

A website PIANO, which stands for **p**seudouridine site **i**dentification **a**nd fu**n**ctional ann**o**tation, was built for the convenience of academic users. Hyper Text Markup Language (HTML), Cascading Style Sheets (CSS), and Hypertext Preprocessor (PHP) were used to construct the PIANO web interface. This included a database containing 4,303 experimentally validated Ψ sites reported from four different high-throughput Ψ profiling techniques, which is so far the most complete collection of Ψ in humans. Among those experimentally validated Ψ sites, we found Ψ was distributed most often along coding DNA sequence and 3’UTR, but it was relatively rare in 5’UTR (**Supplementary Figure S2**). Secondly, a web server for putative Ψ-site identification from the user-defined genomic ranges or provided FASTA sequences (detailed in **Figure 2**) was used. The help document of the PIANO web server is provided in the **Supplementary Materials**. Both experimentally validated Ψ sites and the predicted putative Ψ sites are functionally annotated with various post-transcriptional regulations to unveil potential functional mechanism concerning Ψ. The data and prediction results may be conveniently downloaded and visualized with web browser. The PIANO website is freely accessible from: http://piano.rnamd.com.

## Conclusion

With recent advancements that unveiled various biomolecular functions of Ψ under different biological contexts, Ψ starts to capture broader interests of the scientific community (Schwartz et al., 2014; Carlile et al., 2014; Li X, et al., 2015; Karijolich et al., 2015; Dominissini et al., 2016; Penzo et al., 2017; Guzzi et al., 2018; Adachi et al., 2018; Shaheen et al., 2019). To date, a number of high-throughput approaches have been developed for profiling the transcriptome-wide distribution of Ψ (Adachi et al., 2019), including Pseudo-seq (Carlile et al., 2014), Ψ-seq (Schwartz et al., 2014), PSI-seq (Lovejoy et al., 2014), CeU-seq (Li X, et al., 2015), and RBS-seq (Khoddami et al., 2019). These technologies all reported the widespread occurrence of Ψ on mRNA and lncRNA in human cells. Four Ψ site predictors have been built, including PseUI (He et al., 2018), XG-PseU (Liu et al., 2019), iRNA-PseU (Chen et al., 2016), and PPUS (Li Y.H, et al., 2015); however, all of them are based on sequence-derived features only without considering other genomic features that may contribute to the prediction and thus limited their performance.

Here, by integrating 42 genomic features together with conventional sequence-derived features, we have developed the (so far) most accurate Ψ-site predictor. Our new method (PIANO) substantially outperformed competing approaches when using four different Ψ profiling protocols as the benchmarks (with 0.24 and 0.12 improvement in terms of AUC under full transcript and mature mRNA modes, respectively) and supports functional annotation for the putative Ψ sites. A web site—PIANO—was also developed, including (1) a database hosting currently the largest collection of 4,303 experimentally validated human Ψ sites; and (2) a web server enabling the prediction of novel Ψ sites from given genomic ranges or FASTA sequences. Users may query and download their predicted results with clear and simple instructions (see **Supplementary Materials**). The scripts used to generate genomic and sequence features considered in PIANO’s framework, the training and testing data, and datasets related to the construction of the PIANO database were provided in the download page of PIANO website. In conclusion, our work will serve as a useful resource for researchers who are interested in Ψ and its role concerning various post-transcriptional regulations.

Nevertheless, it is worth noting that there exist significant discrepancies in the Ψ sites reported by different technologies (Zaringhalam and Papavasiliou, 2016; Adachi et al., 2018). Although the discrepancy may be due to the context-specificity of pseudouridylation and technology preferences, our PIANO predictor achieved reasonable consensus with all the four high-throughput profiling Ψ techniques; Ψ is, however, considered as the most prevalent mRNA modifications (Meyer and Jaffrey, 2017) with an estimated Ψ/U ratio of 7–9% (Jacob et al., 2017). Currently, only a small number of Ψ sites have been reported; we are therefore not able to calculate a reasonable number for the real-life estimate of class imbalance. This may due to the limited detection power of existing experimental approaches. With an estimated real-life Ψ/U ratio as 8%, we can expect at least 10 times the number of negative sites. Under this assumption, we tested the stability of our method by assigning 1:10 and 1:1 positive-to-negative ratio for the training and testing data. The result showed that the performance generated by the 1:10 class were more stable than the 1:1 class (**Supplementary Figure S3**). We further calculated the value of FDR, FPR, and TPR in this setting, using different LRs as cutoff (**Supplementary Table S7**). To sum up, we cannot rule out the possibility of experimental bias, and the training data (gold standard data) may be further optimized in the future as more experimental evidence is accumulated. To make the PIANO method more practically useful, the predictor should be used by combining with other experimental evidence and knowledge, e.g., the Us within a binding site of PUS. The performance of PIANO method is much better than all existing approaches, and it can provide the most reliable putative Ψ sites for users.

## Data Availability Statement

Publicly available datasets were analyzed in this study. This data can be found here: GSE60047, GSE58200, GSE63655, GSE90963.

## Author Contributions

KC, JM, GL, and JS initialized the project. KC and BS designed the research plan. ZW constructed the genomic features considered in human Ψ site prediction. BS performed the development of the Ψ site web server. YT and BS built the website. BS and KC drafted the manuscript. All authors read, critically revised, and approved the final manuscript.

## Funding

This work has been supported by the National Natural Science Foundation of China [31671373]; XJTLU Key Program Special Fund [KSF-T-01].

## Conflict of Interest

The authors declare that the research was conducted in the absence of any commercial or financial relationships that could be construed as a potential conflict of interest.
