# Supplementary material for: PIANO: A Web Server for Pseudouridine-Site (Ψ) Identification and Functional Annotation
Source: Front Genet. 2020 Mar 12;11:88. doi: 10.3389/fgene.2020.00088 (PMC7080813; doi:10.3389/fgene.2020.00088)
Supplement: Supplementary file 1 [file DataSheet_1.docx]

**Supplementary Tables and Figures**

**Table S1 Genome-derived features used for human Ψ site prediction**

| ID | Name | Description | Note |
| --- | --- | --- | --- |
| 1 | UTR5 | 5' UTR | Dummy variables indicating whether the site is overlapped to the topological region on the major RNA transcript. |
| 2 | UTR3 | 3' UTR |  |
| 3 | cds | Coding sequence |  |
| 4 | Stop_codons | stop codons flanked by 100bp |  |
| 5 | Start_codons | start codons flanked by 100bp |  |
| 6 | TSS | downstream 100bp of TSS |  |
| 7 | TSS_A | downstream 100bp of TSS on A |  |
| 8 | exon_stop | exons containing stop codons |  |
| 9 | alternative_exon | alternative exons |  |
| 10 | constitutive_exon | constitutive exons |  |
| 11 | internal_exon | Internal exons |  |
| 12 | long_exon | long exons (exon length >= 400bp) |  |
| 13 | last_exon | 5’ last_exon |  |
| 14 | last_exon_400bp | 5’ 400bp of the last exons |  |
| 15 | last_exon_sc400 | 5’ 400bp of the last exons containing stop codons |  |
| 16 | intron | intron |  |
| 17 | pos_cds | relative position on coding sequence | Relative position on the region |
| 18 | pos_UTR5 | relative position on 5'UTR |  |
| 19 | pos_UTR3 | relative position on 3'UTR |  |
| 20 | pos_exons | relative position on exon |  |
| 21 | length_UTR5 | 5'UTR length | The region length in bp. |
| 22 | length_UTR3 | 3'UTR length |  |
| 23 | length_gene_ex | mature transcript length |  |
| 24 | length_cds | Coding sequence length |  |
| 25 | length_gene_full | full transcript length |  |
| 26 | dist_sj_5_p2000 | distance to the 5' splicing junction | Nucleotide distances toward the splicing junctions or the nearest neighboring sites. |
| 27 | dist_sj_3_p2000 | distance to the 3' splicing junction |  |
| 28 | PC_1bp | phastCons scores of the nucleotide | Scores related to evolutionary conservation |
| 29 | PC_101bp | average phastCons scores within the flanking 50bp |  |
| 30 | FC_1bp | fitCons scores of the nucleotide |  |
| 31 | FC_101bp | average fitCons scores within the flanking 50bp region |  |
| 32 | struc_hybridize | Predicted RNA hybridized region | RNA secondary structures |
| 33 | struc_loop | Predicted RNA loop region |  |
| 34 | sncRNA | sncRNA | Genomic properties |
| 35 | lncRNA | lncRNA |  |
| 36 | HK_genes | housekeeping genes |  |
| 37 | isoform_num | Number of isoform |  |
| 38 | exon_num | Number of exon |  |
| 39 | HNRNPC_eCLIP | eCLIP data of HNRNPC RNA binding sites | Attributes of the genes or transcripts |
| 40 | Verified_miRtargets | miRNA targeted sites verified by experiment |  |
| 41 | TargetScan | Predicted miRNA targeted sites by TargetScan |  |
| 42 | miR_targeted_genes | miRNA targeted genes |  |

**Table S2 Data used for** **Post-transcriptional regulation association analysis**

|  | Category | Number |
| --- | --- | --- |
| Association Analysis Part I:  RBP binding sites | RBP binding sites from POSTAR2 | 28,898,046 |
| Association Analysis Part II:  miRNA-RNA interactions | miRNA-mRNA interaction from miRanda | 948,053 |
|  | miRNA-lncRNA interaction from startBase2 | 24,086 |
| Association Analysis Part III:  Splicing sites | Canonical splice sites (GT-AG) | 442,553 |

**Table S3 Human Ψ site prediction using different feature sets**

| Mode | Testing  method | Predictor | Base-resolution Technique and Dataset ID | | | | | | Average |
| --- | --- | --- | --- | --- | --- | --- | --- | --- | --- |
|  |  |  | Ψ-Seq | RBS-Seq | CeU-Seq | | | |  |
|  |  |  | H1 | H2 | H3 | H4 | H5 | Average |  |
| Full  Transcript | Cross  validation | PIANO | 0.966 | 0.953 | 0.965 | 0.965 | 0.971 | 0.967 | **0.964** |
|  |  | iRNA-PseU | 0.814 | 0.767 | 0.814 | 0.807 | 0.795 | 0.805 | 0.799 |
|  |  | PPUS | 0.809 | 0.756 | 0.807 | 0.802 | 0.807 | 0.805 | 0.796 |
|  |  | PesUI | 0.765 | 0.737 | 0.774 | 0.765 | 0.758 | 0.766 | 0.760 |
|  | Independent  Dataset | PIANO | 0.957 | 0.978 | 0.977 | 0.972 | 0.794 | 0.914 | **0.936** |
|  |  | iRNA-PseU | 0.697 | 0.727 | 0.794 | 0.736 | 0.633 | 0.721 | 0.717 |
|  |  | PPUS | 0.700 | 0.721 | 0.796 | 0.740 | 0.636 | 0.724 | 0.719 |
|  |  | PesUI | 0.631 | 0.710 | 0.637 | 0.625 | 0.569 | 0.610 | 0.634 |
| mature  mRNA | Cross  validation | PIANO | 0.866 | 0.870 | 0.852 | 0.866 | 0.864 | 0.861 | **0.863** |
|  |  | iRNA-PseU | 0.772 | 0.776 | 0.751 | 0.771 | 0.771 | 0.764 | 0.768 |
|  |  | PPUS | 0.776 | 0.779 | 0.755 | 0.775 | 0.775 | 0.768 | 0.772 |
|  |  | PesUI | 0.700 | 0.708 | 0.682 | 0.701 | 0.700 | 0.694 | 0.698 |
|  | Independent  Dataset | PIANO | 0.859 | 0.770 | 0.856 | 0.867 | 0.868 | 0.864 | **0.844** |
|  |  | iRNA-PseU | 0.753 | 0.582 | 0.759 | 0.761 | 0.761 | 0.760 | 0.723 |
|  |  | PPUS | 0.749 | 0.575 | 0.756 | 0.757 | 0.758 | 0.757 | 0.719 |
|  |  | PesUI | 0.666 | 0.651 | 0.649 | 0.648 | 0.660 | 0.652 | 0.655 |

**Note**: PIANO achieved substantial improvements compared to existing approaches in cross-validation or when tested on an independent dataset.

**Table S4. Human Ψ site prediction evaluated on an independent technique**

| **Predictor** | **Full transcript model** | **Mature mRNA model** |
| --- | --- | --- |
| PIANO | 0.972 | 0.857 |
| iRNA-PseU | 0.708 | 0.751 |
| PPUS | 0.705 | 0.748 |
| PesUI | 0.585 | 0.639 |

**Note**: Datasets generated from Ψ-Seq, RBS-Seq and CeU-Seq were used for training; while the performance was evaluated on dataset generated from a different technique (Pseudo-seq). PIANO achieved substantial improvements compared to existing approaches under both full transcript and mature mRNA mode

**Table S5. Performance evaluation of PIANO by separating training and testing dataset between cell types**

| **Method** | **Algorithm** | **Independent test** | | | | | | | | | | | | |
| --- | --- | --- | --- | --- | --- | --- | --- | --- | --- | --- | --- | --- | --- | --- |
|  |  | **Full transcript model** | | | | | | **Mature mRNA model** | | | | | | |
|  |  | **AUROC** | **AUPRC** | **ACC** | **Sp** | **Sn** | **MCC** | **AUROC** | **AUPRC** | **ACC** | **Sp** | **Sn** | **MCC** | |
| PINAO | SVM | 0.944 | 0.916 | 0.812 | 0.901 | 0.851 | 0.707 | 0.799 | 0.793 | 0.741 | 0.679 | 0.705 | 0.416 |  |
|  | RF | 0.897 | 0.879 | 0.765 | 0.803 | 0.782 | 0.565 | 0.784 | 0.773 | 0.790 | 0.666 | 0.711 | 0.439 |  |
|  | GLM | 0.729 | 0.752 | 0.722 | 0.708 | 0.715 | 0.430 | 0.769 | 0.771 | 0.698 | 0.674 | 0.685 | 0.370 |  |
|  | DT | 0.719 | 0.412 | 0.660 | 0.787 | 0.690 | 0.411 | 0.620 | 0.367 | 0.619 | 0.578 | 0.594 | 0.192 |  |
| iRNA | SVM | 0.662 | 0.633 | 0.658 | 0.590 | 0.615 | 0.239 | 0.682 | 0.692 | 0.673 | 0.599 | 0.626 | 0.262 |  |
|  | RF | 0.681 | 0.684 | 0.640 | 0.572 | 0.595 | 0.200 | 0.688 | 0.705 | 0.694 | 0.588 | 0.621 | 0.262 |  |
|  | GLM | 0.645 | 0.608 | 0.632 | 0.582 | 0.601 | 0.208 | 0.662 | 0.650 | 0.639 | 0.587 | 0.607 | 0.219 |  |
|  | DT | 0.624 | 0.413 | 0.581 | 0.597 | 0.588 | 0.177 | 0.697 | 0.299 | 0.642 | 0.642 | 0.641 | 0.283 |  |
| PPUS | SVM | 0.655 | 0.620 | 0.672 | 0.602 | 0.628 | 0.265 | 0.654 | 0.677 | 0.635 | 0.572 | 0.594 | 0.198 |  |
|  | RF | 0.649 | 0.679 | 0.630 | 0.568 | 0.589 | 0.188 | 0.662 | 0.680 | 0.671 | 0.565 | 0.595 | 0.211 |  |
|  | GLM | 0.672 | 0.614 | 0.677 | 0.614 | 0.638 | 0.284 | 0.639 | 0.640 | 0.617 | 0.565 | 0.584 | 0.175 |  |
|  | DT | 0.562 | 0.413 | 0.537 | 0.556 | 0.545 | 0.091 | 0.550 | 0.430 | 0.562 | 0.541 | 0.549 | 0.101 |  |
| PseUI | SVM | 0.799 | 0.793 | 0.741 | 0.679 | 0.705 | 0.416 | 0.744 | 0.747 | 0.729 | 0.636 | 0.670 | 0.353 |  |
|  | RF | 0.784 | 0.773 | 0.790 | 0.666 | 0.711 | 0.439 | 0.769 | 0.760 | 0.747 | 0.621 | 0.666 | 0.359 |  |
|  | GLM | 0.769 | 0.771 | 0.698 | 0.674 | 0.685 | 0.370 | 0.715 | 0.711 | 0.696 | 0.630 | 0.656 | 0.319 |  |
|  | DT | 0.620 | 0.367 | 0.619 | 0.578 | 0.594 | 0.192 | 0.603 | 0.466 | 0.650 | 0.565 | 0.589 | 0.195 |  |

**Note:** Datasets generated from HEK293T were used for training, while datasets generated from a different cell type Hela were used for independent testing. To gain a better presentation of the compared results, we further tested the performance of PIANO and other compared methods using different algorithms. We reproduced the machine learning scaffold of the compared methods by using each of their sequence-based encoding methods, and the predictors are trained and tested with the same training and testing data used in our study.

SVM: Support Vector Machine; RF: Random Forest; GLM: generalized linear model; DT: decision tree.

**Table S6. Performance evaluation of PIANO by using independent testing dataset with 1:10 positive to negative ratio**

| **Method** | **Independent test** | | | | | |
| --- | --- | --- | --- | --- | --- | --- |
|  | **Mature mRNA model** | | | | | |
|  | **AUROC** | **AUPRC** | **Sn** | **Sp** | **ACC** | **MCC** |
| PIANO | 0.792 | 0.369 | 0.618 | 0.767 | 0.754 | 0.250 |
| PPUS | 0.665 | 0.161 | 0.509 | 0.713 | 0.694 | 0.138 |
| iRNA | 0.648 | 0.144 | 0.513 | 0.704 | 0.687 | 0.135 |
| PseUI | 0.725 | 0.295 | 0.564 | 0.762 | 0.744 | 0.211 |

**Note:** Datasets generated from HEK293T were used for training, while datasets generated from a different cell type Hela were used for independent testing with 1:10 positive to negative ratio.

**Table S7. Value of FDR, FPR, and TPR at different LRs as cutoff**

| **Likekihood ratio (LR)** | **FPR** | **TPR** | **FDR**  **(given 8% Ψ/U ratio)** |
| --- | --- | --- | --- |
| 1 | 0.287 | 0.909 | 0.784 |
| 2 | 0.127 | 0.782 | 0.652 |
| 3 | 0.064 | 0.582 | 0.557 |
| 4 | 0.025 | 0.473 | 0.382 |
| 5 | 0.009 | 0.364 | 0.223 |
| 6 | 0.007 | 0.291 | 0.223 |
| 7 | 0.004 | 0.236 | 0.150 |
| 8 | 0.004 | 0.182 | 0.187 |
| 9 | 0.002 | 0.182 | 0.103 |
| 10 | 0.002 | 0.182 | 0.103 |
| 11 | 0.002 | 0.182 | 0.103 |
| 12 | 0.002 | 0.164 | 0.113 |
| 13 | 0.002 | 0.164 | 0.113 |
| 14 | 0 | 0.164 | < 0.113 |

**Note:** To validate the performance of the predictive model trained by data from HEK293T cell line, datasets from a different cell type (Hela) were used for independent testing. The value of FDR, FPR, and TPR was calculated by using different LRs as cutoff, the false discovery rate (FDR) was calculated given an estimate of 8% Ψ/U ratio. The likelihood ratio (LR) of a Ψ site was calculated to estimate the probability of Ψ RNA methylation as mentioned in the METHOD section.


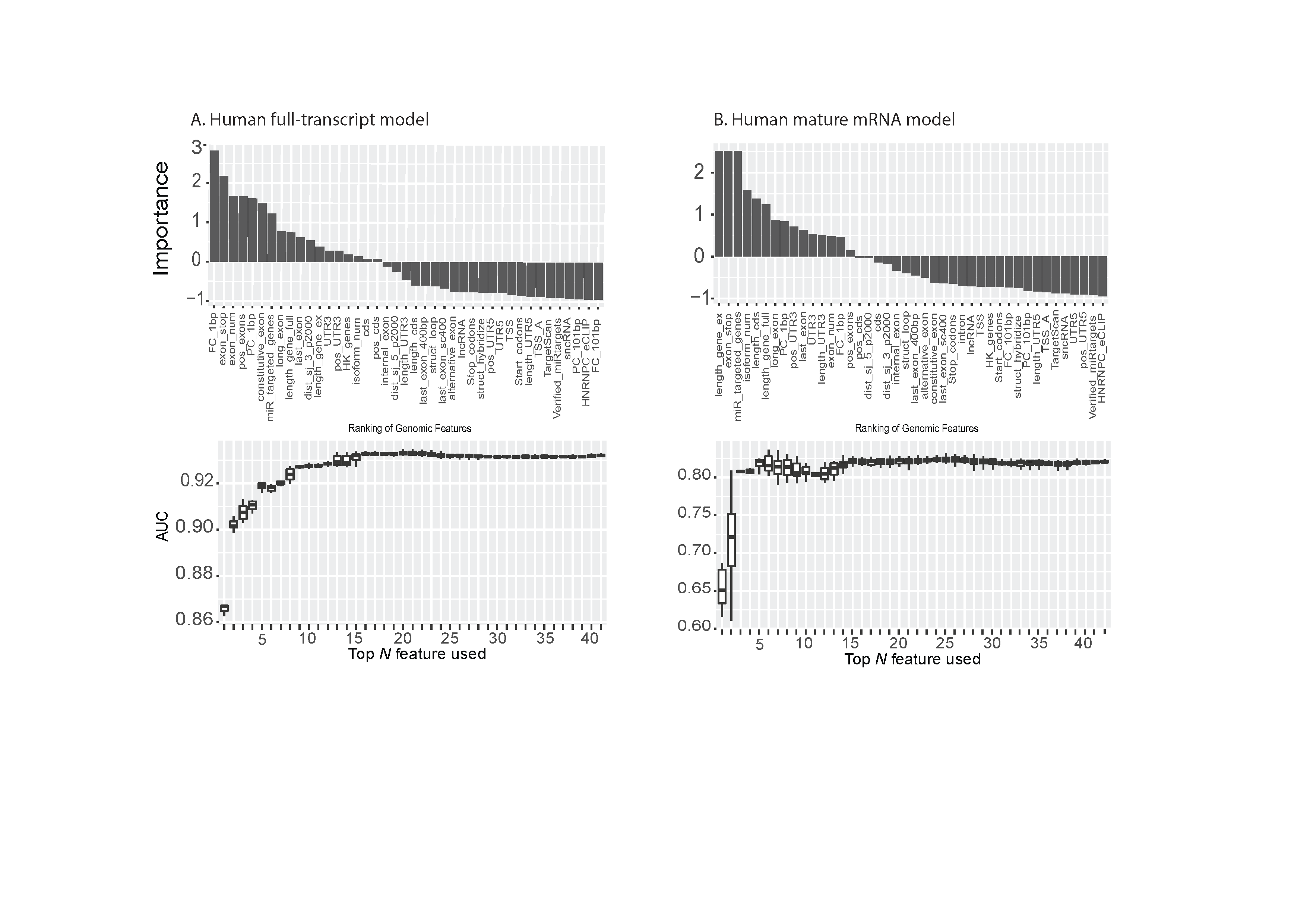


**Figure S1.** Feature selection of the genome-derived features for Ψ site prediction. Top 17 and 20 genomic features were used in further prediction under human full transcript model **(Figure S1A)**, and mature mRNA model **(Figure S1B)** respectively. The importance of each feature under different models was assessed by the Perturb method. The performance (AUC) was evaluated using 5-fold cross-validation. The datasets H2-H5 were used as the training data for human.

**Figure S2. The location of 4303 experimentally validated Ψ sites within transcripts.** Ψ was distributed most along coding DNA sequence and 3’UTR, but was relatively rare in 5’UTR.

**Figure S3. The stability comparison between performances generated from 1:1 and 1:10 class.** In PIANO method, we randomly selected 10 negative sites for each of the positive site. In order to balance the positive-to-negative ratio, the negative sites were then randomly split into 10 subsets to generate 10 separate predictors with 1:1 positive-to-negative ratio, and their performance were averaged (1:10 class). To test the stability of this method, we repeated this process 10 times, and recorded each round of their averaged performances. We then randomly selected 1 negative site for each positive site and evaluated its performance (1:1 class). We also repeated this 1:1 ratio for 10 times. The result showed the performance generated by 1:10 class were more stable than 1:1 class, indicating the stability of the testing method used in PIANO.
